# Analysis and Optimization of a Novel 2-D Magnet Array with Gaps and Staggers for a Moving-Magnet Planar Motor

**DOI:** 10.3390/s18010124

**Published:** 2018-01-04

**Authors:** Yang Wang, Xuedong Chen, Xin Luo, Lizhan Zeng

**Affiliations:** State Key Laboratory of Digital Manufacturing Equipment and Technology, Huazhong University of Science and Technology, Wuhan 430074, China; wyyhust@hust.edu.cn (Y.W.); chenxd@mail.hust.edu.cn (X.C.); mexinluo@hust.edu.cn (X.L.)

**Keywords:** 2-D magnet array, high-order harmonics, planar motor, optimization

## Abstract

This paper presents a novel 2-D magnet array with gaps and staggers, which is especially suitable for magnetically levitated planar motor with moving magnets. The magnetic flux density distribution is derived by Fourier analysis and superposition. The influences of gaps and staggers on high-order harmonics and flux density were analyzed, and the optimized design is presented. Compared with the other improved structures based on traditional Halbach magnet arrays, the proposed design has the lowest high-order harmonics percentage, and the characteristics of flux density meet the demand of high acceleration in horizontal directions. It is also lightweight and easy to manufacture. The proposed magnet array was built, and the calculation results have been verified with experiment.

## 1. Introduction

With the rapid development of the Micro-Electro-Mechanical System (MEMS) and semiconductor industry, a magnetically levitated planar motor is becoming an optimal choice to meet the requirements of high speed, high precision, and high vacuum in lithography equipment and various microscopes [1,2,3]. A magnetically levitated planar motor generally contains a 2-D magnet array and an ironless coil array, and planar motors can be classified into moving-coil type and moving-magnet type according to different moving part. Moving-coil type planar motors have the disadvantages of cable interference and inconvenient cooling system. Oppositely, planar motors with moving magnets can avoid these problems completely and thus have the potential for higher precision [4].

As the mover of a planar motor is magnetically levitated and driven by Lorentz force, the magnetic field produced by the magnet array plays a crucial role in determining the speed and accuracy of the planar motor. The flux density determines the thrust force, acceleration, and max speed. The high-order harmonics greatly influence positioning accuracy because the high-order harmonics are ignored to save time in real-time control and the simplification leads to force ripple. There are two ways to reduce the force ripple: one is to compensate high-order harmonics with a control method, and the other is to optimize the structure of the magnet array for lower high-order harmonics. Obviously, the latter is better because it can fundamentally restrain the force ripple.

Much research has been done to optimize the traditional Halbach magnet array for low high-order harmonics and large flux density. For instance, Trumper et al. [5] investigated different magnet array topologies and proposed an ideal Halbach magnet array. Min et al. [6] proposed a 2-D magnet array with 45° magnetized magnets. Peng et al. [7] proposed a new Halbach magnet array with trapezoidal magnets, and Liu et al. [8] used hexagon magnets as the horizontal magnetized magnets. Zhang et al. [9] used a two-layer magnet array for low high-order harmonics and large flux density. Although these designs can reduce high-order harmonics, the structures are too complex to manufacture. Furthermore, as the density of NdFeB permanent magnet is nearly three times that of mounting plates, which are usually aluminum, the magnet array for the planar motor with moving magnets should be lightweight to achieve high acceleration and low energy consumption.

In this paper, we propose an easy-to-manufacture 2-D magnet array with gaps and staggers to reduce the high-order harmonics and self-weight. As the 2-D magnet array is a periodic structure in the horizontal directions, Fourier analysis was used to model the magnetic field. Compared with the time-consuming finite element method, the analytical method based on Fourier analysis can determine the field efficiently and accurately, and it is more suitable for further analysis and optimization. The magnet array was then separated into two sub-arrays for simplification of analysis, and the flux density distribution of the total magnet array was derived by superposition. Afterwards, the influences of gaps and staggers on the high-order harmonics and flux density were obtained, and a set of structural parameters of the proposed design were calculated by minimizing the high-order harmonics. The calculation shows that the high-order harmonics percentage of the novel 2-D magnet array is much lower than that of other designs and that the *^m^z*-component of flux density is larger than that of traditional Halbach magnet arrays. At last, the proposed magnet array was built, and the flux density distribution was measured to validate the theoretical results.

## 2. Materials and Methods

**Figure 1:**
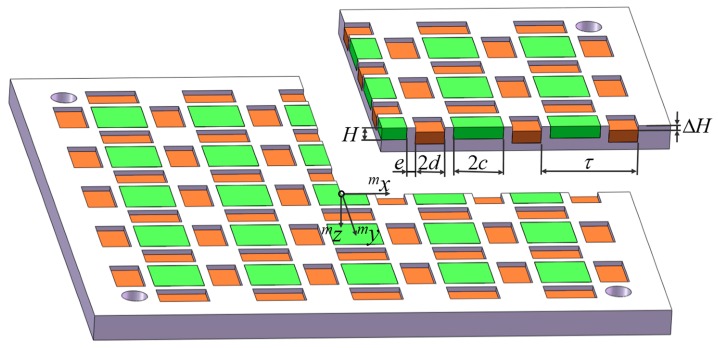
Sectional view of the novel magnet array with gaps and staggers.

Figure 1 shows a sectional view of the novel magnet array with gaps and staggers. There are gaps between neighboring vertical and horizontal magnetized magnets, and the mounting heights of these two kinds of magnets are different. The gap width is *e*, and the mounting height difference is ∆H. *τ* is the pole pitch, and *H* is the height of all magnets. *c* is the half width of the vertical magnetized magnets, while *d* is the half width of the horizontal magnetized magnets. The coordinate system center is set in the center of a vertical magnetized magnet’s top surface.

### 2.1. Modeling

**Figure 2:**
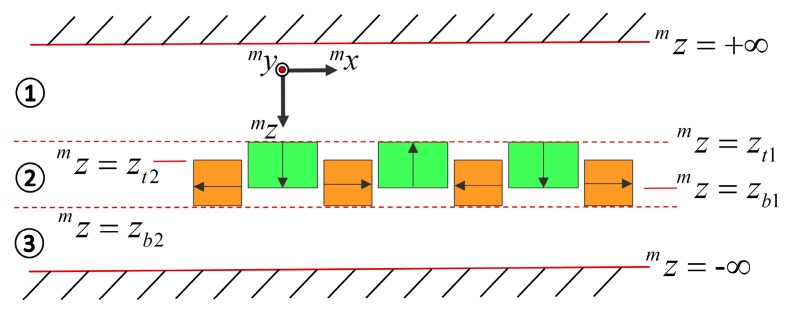
Analytical model of the novel magnet array.

As shown in Figure 2, the space was divided into three regions to derive the flux density distribution of the magnet array. The arrows in the figure denote the magnetization direction of the permanent magnets from the S-pole to the N-pole, and the magnetic flux density distribution strengthens above the magnet array. As NdFeB permanent magnets have a relative permeability of 1.03–1.05, which is close to the relative permeability of air, the empties of the magnet array could be neglected in Region 2. Equations (1)–(4) are valid for Regions 1 and 3, while Equations (5)–(9) are applicable to Region 2.

(1) ∇2φnm=0

(2) H→nm=−∇φnm

(3) B→nm=μ0H→nm

(4) ∇·B→nm=0

where *n* takes 1 or 3, representing Region 1 or Region 3.

(5) ∇2φ2m=∇·M→mμr

(6) H→2m=−∇φ2m

(7) ∇×H→2m=0

(8) B→2m=μ0μrH→2m+μ0M→m

(9) ∇·B→2m=0.

According to Figure 2, the boundary conditions can be expressed as follows:

(10) φ1m(zm=+∞)=0

(11) φ3m(zm=−∞)=0

(12) H1xm(zm=zti)=H2xm(zm=zti)

(13) H1ym(zm=zti)=H2ym(zm=zti)

(14) H2xm(zm=zbj)=H3xm(zm=zbj)

(15) H2ym(zm=zbj)=H3ym(zm=zbj)

(16) B1zm(zm=zti)=B2zm(zm=zti)

(17) B2zm(zm=zbj)=B3zm(zm=zbj)

where *i* takes 1 or 2; meanwhile, *j* takes 2 or 1, because ∆H may be positive or negative.

In order to simplify the calculation, two sub-arrays were first analyzed separately. One sub-array contains the vertical magnetized magnets, while the other contains the horizontal magnetized magnets. The magnetization vector function of the sub-array with vertical magnetized magnets can be expressed as a Fourier series in Equation (18) and that of the sub-array with horizontal magnetized magnets can be expressed as Equation (19). The detailed deriving process is shown in Appendix A.

(18) M1→m=Brμ0∑k=1∞∑l=1∞[00akalcoskωx⋅coslωy]

(19) M2→m=Brμ0∑k=1∞∑l=1∞[bkalsinkωx⋅coslωyakblcoskωx⋅sinlωy0]

where *k* and *l* are the harmonic numbers for the *^m^x*- and *^m^y*-directions respectively, and $b_{k}=\frac{4}{k\pi }\sin(\frac{k\pi }{2})\sin(k\omega d)$, $a_{l}=\frac{4}{l\pi }\sin(\frac{l\pi }{2})\cos[l\omega (d+e)]$, $a_{k}=\frac{4}{k\pi }\sin(\frac{k\pi }{2})\cos[k\omega (d+e)]$, $b_{l}=\frac{4}{l\pi }\sin(\frac{l\pi }{2})\sin(l\omega d)$.

The flux density distribution can be obtained by calculating differential equations by employing all the equations above [10,11]. As a result, the magnetic flux density distribution of the sub-array with vertical magnetized magnets can be expressed as Equation (20), while the expression of the sub-array with horizontal magnetized magnets is Equation (21). The detailed deriving process is shown in Appendix B.

(20) $${\vec{B}}_{1V}=\frac{1}{2}B_{r}\sum_{k=1}^{\infty }\sum_{l=1}^{\infty }\left(e^{-\lambda z_{b1}}-e^{-\lambda z_{t1}}\right)e^{\lambda z}\cdot \left[\begin{array}{c} \frac{a_{k}a_{l}\sqrt{k^{2}+l^{2}}}{k^{2}+l^{2}}k\sin k\omega x\cos l\omega y\\ \frac{a_{k}a_{l}\sqrt{k^{2}+l^{2}}}{k^{2}+l^{2}}l\cos k\omega x\sin l\omega y\\ \begin{array}{l} -a_{k}a_{l}\cos k\omega x\cos l\omega y \end{array} \end{array}\right]$$

(21) $${\vec{B}}_{1H}=\frac{1}{2}B_{r}\sum_{k=1}^{\infty }\sum_{l=1}^{\infty }\left(e^{-\lambda z_{b2}}-e^{-\lambda z_{t2}}\right)e^{\lambda z}\cdot \left[\begin{array}{c} \frac{E\left(k,l\right)}{k^{2}+l^{2}}k\sin k\omega x\cos l\omega y\\ \frac{E\left(k,l\right)}{k^{2}+l^{2}}l\cos k\omega x\sin l\omega y\\ -\frac{E\left(k,l\right)}{\sqrt{k^{2}+l^{2}}}\cos k\omega x\cos l\omega y \end{array}\right]$$

where $\lambda =\frac{\pi }{\tau }\sqrt{k^{2}+l^{2}}$, and $E(k,l)=b_{k}a_{l}k+a_{k}b_{l}l$.

With the superposition method, the flux density distribution of the total magnet array in Region 1 can be obtained as follows:

(22) $${\vec{B}}_{1}=\frac{1}{2}B_{r}\sum_{k=1}^{\infty }\sum_{l=1}^{\infty }e^{\lambda z}\left[\begin{array}{c} \left[\left(e^{-\lambda z_{b2}}-e^{-\lambda \left(z_{b2}-H\right)}\right)\frac{E\left(k,l\right)}{k^{2}+l^{2}}+\left(e^{-\lambda \left(z_{b2}-\Delta H\right)}-e^{-\lambda \left(z_{b2}-\Delta H-H\right)}\right)\frac{a_{k}a_{l}\sqrt{k^{2}+l^{2}}}{k^{2}+l^{2}}\right]k\sin k\omega x\cos l\omega y\\ \left[\left(e^{-\lambda z_{b2}}-e^{-\lambda \left(z_{b2}-H\right)}\right)\frac{E\left(k,l\right)}{k^{2}+l^{2}}+\left(e^{-\lambda \left(z_{b2}-\Delta H\right)}-e^{-\lambda \left(z_{b2}-\Delta H-H\right)}\right)\frac{a_{k}a_{l}\sqrt{k^{2}+l^{2}}}{k^{2}+l^{2}}\right]l\cos k\omega x\sin l\omega y\\ -\left[\left(e^{-\lambda z_{b2}}-e^{-\lambda \left(z_{b2}-H\right)}\right)\frac{E\left(k,l\right)}{\sqrt{k^{2}+l^{2}}}+\left(e^{-\lambda \left(z_{b2}-\Delta H\right)}-e^{-\lambda \left(z_{b2}-\Delta H-H\right)}\right)a_{k}a_{l}\right]\cos k\omega x\cos l\omega y \end{array}\right].$$

Compared with the magnetic field distribution raised by Jansen [11], this model is more universal. Jansen’s design can be regarded as a special case of Equation (22) where *e* takes 0 and Δ*H* takes 0.

### 2.2. Analysis and Optimization

**Figure 3:**
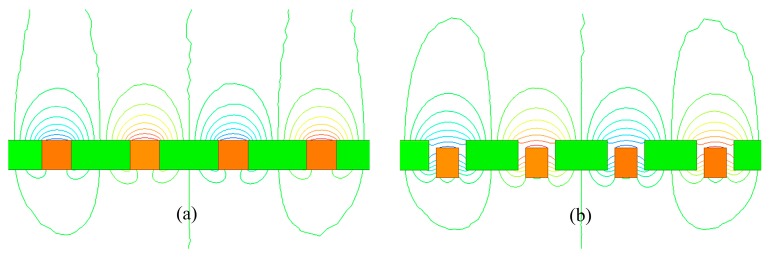
Flux lines distribution. (**a**) The traditional Halbach magnet array; (**b**) the novel magnet array with gaps and staggers.

As the magnetization vectors are weakened in the gaps between the magnets, the novel magnet array is more similar to the ideal Halbach array [5] than the traditional Halbach magnet array. Moreover, the staggers can pull down the near-surface flux lines that contain large high-order harmonics percentage as shown in Figure 3. Therefore, this novel magnet array has the potential for lower high-order harmonics compared with the traditional Halbach magnet array.

Based on the derived magnetic flux density distribution in Equation (22), the gap ratio (*e/τ*) and stagger ratio (∆*H*/*H*) can be analyzed for optimization. A square plane was chosen to calculate the maximum value of high-order harmonics percentage. The plane is 5 mm above the top of the novel magnet array, and the side length of the square takes *τ*/2. A 20 × 20 point matrix (*P_mn_*) was chosen in the square for calculation. As the magnet array is exactly symmetrical in the *^m^x*- and *^m^y*-directions and the component of flux density distribution in the *^m^z*-direction is similar with those of the *^m^x*- and *^m^y*-directions, we used the *^m^x*-component to write the evaluation function, which is as follows:

(23) $$F(e/\tau ,\Delta H/H,d/\tau )=\max(\left|\frac{B_{1}{}_{x}(P_{mn})-B_{1}{{}_{x}}^{1,1}(P_{mn})}{B_{1}{}_{x}(P_{mn})}\right|)$$

where $P_{mn}(0.025m\tau ,0.025n\tau ,\min(z_{t1},z_{t2})-0.005)$, *B*_1*x*_ is the total flux density containing the first harmonic and high-order harmonics, and *B*_1*x*_^1,1^ is the first harmonic.

When *k* > 20 or *l* > 20, the high-order harmonics are much smaller than the geomagnetic field (about 6 × 10^−5^ T) [6]. Therefore, the harmonic components can be ignored if any one of the harmonic numbers (*k* or *l*) is more than 20 when calculating the flux density.

**Figure 4:**
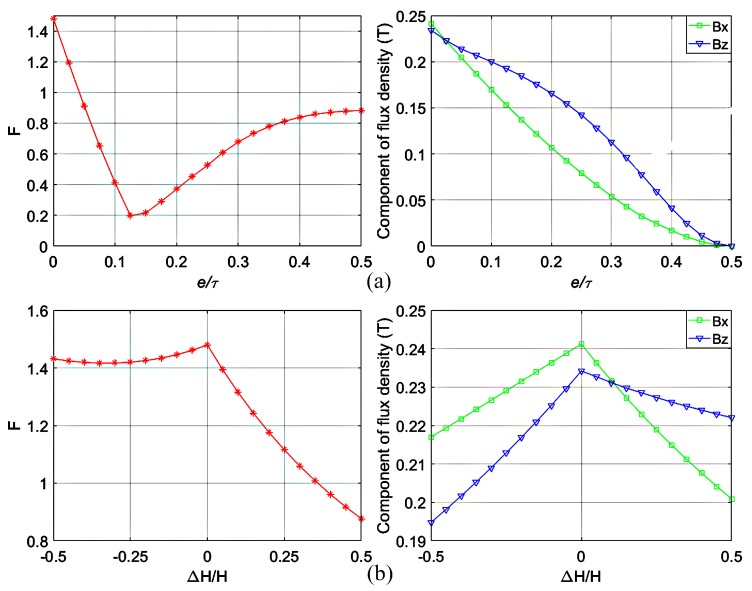
The influences on magnetic field characteristics (**a**) by varying *e*/*τ* when *∆H*/*H* takes 0, and the width ratio of two kinds of magnets (*c*/*d*) kept as 0.68:0.32 and (**b**) by varying *∆H*/*H* when *e*/*τ* takes 0.

The fixed parameters of the magnet array are listed in Table 1. As F presents the high-order harmonics percentage, a lower value is better. By varying the gap ratio and the stagger ratio respectively, we could obtain the influences on F and the maximum values of flux density components in the *^m^x*- and *^m^z*-directions. Figure 4 shows that the novel magnet array with small gaps and staggers can decrease high-order harmonics significantly with a slight decrease in flux density, and the high-order harmonics intensity is more sensitive to gap ratio than to stagger ratio. If the gap ratio is too large, not only does the flux density decrease too much but the high-order harmonics percentage also increases. As the flux density decreases too much with big stagger ratio, the constraint of stagger ratio is set as −0.5 to 0.5. This analysis provides constraints of variables for optimization as shown in Table 2.

Using F as the objective function, an optimized design can be obtained with the genetic algorithm. The max generation was set at 100, while the population size was set at 30. Thus, the minimum of the objective function can be obtained when the gap ratio takes 0.091, the stagger ratio takes 0.374, and the *d*/*τ* takes 0.15. The minimum value of F is 0.1249, while that of Jansen’s is 1.4797.

## 3. Results and Discussion

### 3.1. Comparison

According to the optimized variables, the high-order harmonics and flux density of the proposed magnet array can be calculated with Equation (22). Comparisons of this design with others’ are available.

For other optimized magnet arrays, there is no gap between magnets, so the weights are approximately equal to that of Jansen’s design with the same magnet height. However, with the same magnet height, the weight difference is too great in the comparison between this novel magnet array and Jansen’s, thus losing the reference value. In this case, Jansen’s magnets should be thinner for similar weights. The parameters of the proposed design are compared with those of Jansen’s in Table 3.

**Figure 5:**
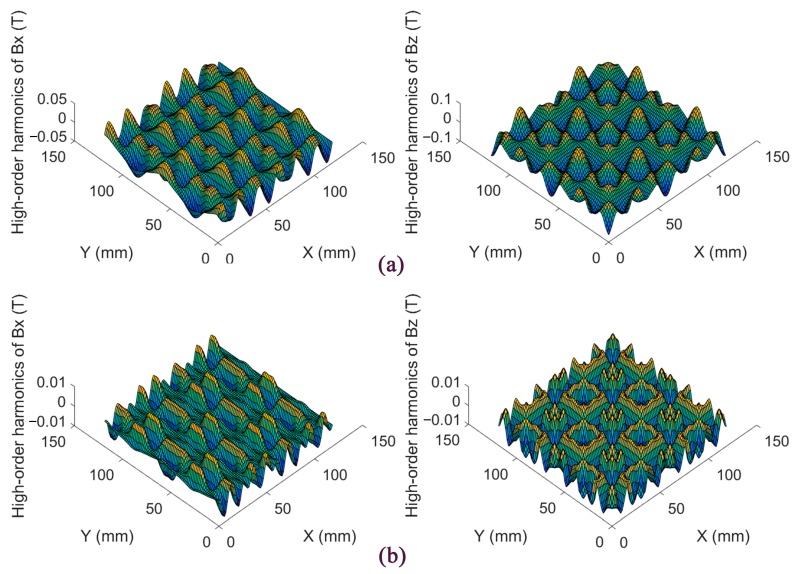
The high-order harmonics in the *^m^x*- and *^m^z*-directions. (**a**) Jansen’s design; (**b**) This proposed design.

The high-order harmonics of the proposed magnet array and Jansen’s design are shown in Figure 5. The peak values of high-order harmonics in this design are 8.36 × 10^−3^ T and 9.44 × 10^−3^ T in the *^m^x*- and *^m^z*-directions, respectively, while those of Jansen’s are correspondingly 3.96 × 10^−2^ T and 6.80 × 10^−2^ T. It is obvious that the novel magnet array decreases the high-order harmonics significantly, and the weight is lighter than Jansen’s by 13.63%.

**Figure 6:**
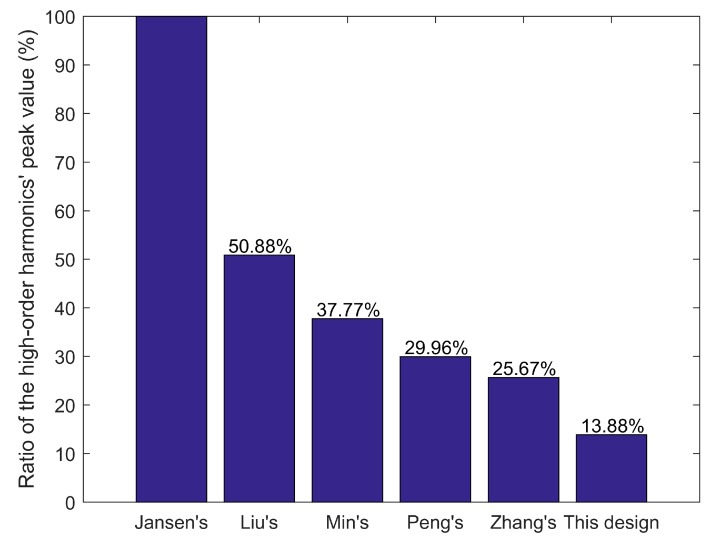
The ratios of high-order harmonics in the *^m^z*-direction compared with Jansen’s design.

Furthermore, Figure 6 shows that the proposed design has the lowest high-order harmonics among the designs as mentioned in the literature [6,7,8,9]. The structure of the proposed design is the simplest and easy to manufacture.

Otherwise, the flux density of this design is stronger than that of Jansen’s by 9.5% in the *^m^z*-direction, which indicates a larger thrust in the horizontal direction. As the flux density in the *^m^x*- and *^m^y*-directions mainly provides levitation force and high acceleration is not required in the vertical direction, a slight decrease in flux density in the *^m^x*-direction is acceptable. 

In summary, the proposed magnet array sharply decreases high-order harmonics in both horizontal and vertical directions and strengthens the thrust in the horizontal direction. These characteristics meet the requirements of magnetically levitated planar motors. Future research will focus on improving the flux density further.

### 3.2. Experiment

**Figure 7:**
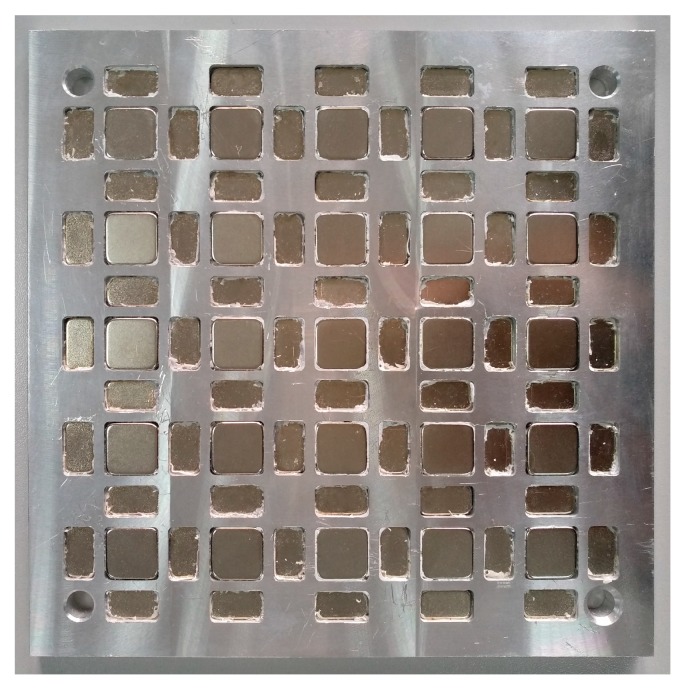
The magnet array built according to the optimized parameters.

In order to validate the theoretical results, an experimental magnet array (as shown in Figure 7) was built according to the optimized structural parameters. The parameters of the magnet array are listed in Table 4.

**Figure 8:**
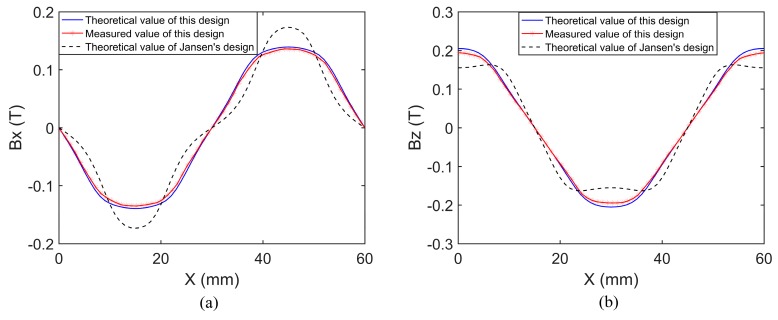
Magnetic flux density distributions of this design and Jansen’s design. (**a**) The *^m^x*-component of flux density; (**b**) the *^m^z*-component of flux density.

The magnetic flux density distribution was measured with the Gauss meter *Lakeshore* 410. Figure 8 shows the measured and theoretical values of this design and those of Jansen’s design in the *^m^x*- and *^m^z*-directions. The magnetic flux density components were measured or calculated along a line 5 mm above the magnet array surface.

It was found that the measured values match well with the theoretical values of the proposed magnet array, so the accuracy of the modeling is convincing. The flux density components of the proposed magnet array are much closer to sine than Jansen’s design in both the *^m^x*- and *^m^z*-directions, which entails lower high-order harmonics. Therefore, the effect of high-order harmonics suppression has been further validated.

## 4. Conclusions

In this paper, a novel magnet array with gaps and staggers is presented and analyzed. The magnetic flux density distribution of this magnet array was derived. The influences on magnetic field of gaps and staggers were revealed, and a set of optimized structural parameters were chosen to decrease high-order harmonics. Compared with other designs in the literature, the proposed magnet array has the lowest high-order harmonics percentage and the simplest structure. The flux density distribution in the *^m^z*-direction is larger than that of the traditional Halbach magnet array, which entails a greater thrust in the horizontal direction. Furthermore, the magnet array is lightweight and suitable for the magnetically levitated planar motor with moving magnets. At last, the proposed magnet array was built, and the calculation conclusions have been verified with experiment.

## Figures and Tables

**Table 1:** Fixed parameters of the novel magnet array.

Fixed Parameters	Value	Unit
Pole pitch (*τ*)	30	mm
Height of the magnets (*H*)	4	mm
Remanence of the permanent magnets (*B_r_*)	1.33	T

**Table 2:** Optimization parameters of the novel magnet array.

Optimization Parameters	Constraints
Gap ratio (*e*/*τ*)	[0, 0.15]
Stagger ratio (∆*H*/*H*)	[−0.5, 0.5]
Width ratio (*d*/*τ*)	[0, 0.35]

**Table 3:** Parameters for the proposed magnet array and Jansen’s.

Parameters	Proposed Magnet Array	Jansen’s
*e*/*τ*	0.091	0
*∆H*/*H*	0.374	0
*d*/*τ*	0.15	0.16
Height of the magnets (mm)	4	3
Weight of the array (g)	431	499

**Table 4:** Parameters of the proposed magnet array.

Parameters	Symbol	Value	Unit
Pole pitch	*τ*	30	mm
Remanence of the permanent magnets	*B_r_*	1.33	T
Width of vertical magnetized magnet	2*c*	15.5	mm
Width of horizontal magnetized magnet	2*d*	9	mm
Width of gap	*e*	2.75	mm
Height of the magnet	*H*	4	mm
Height of the stagger	*∆H*	1.5	mm

**Figure A1:**
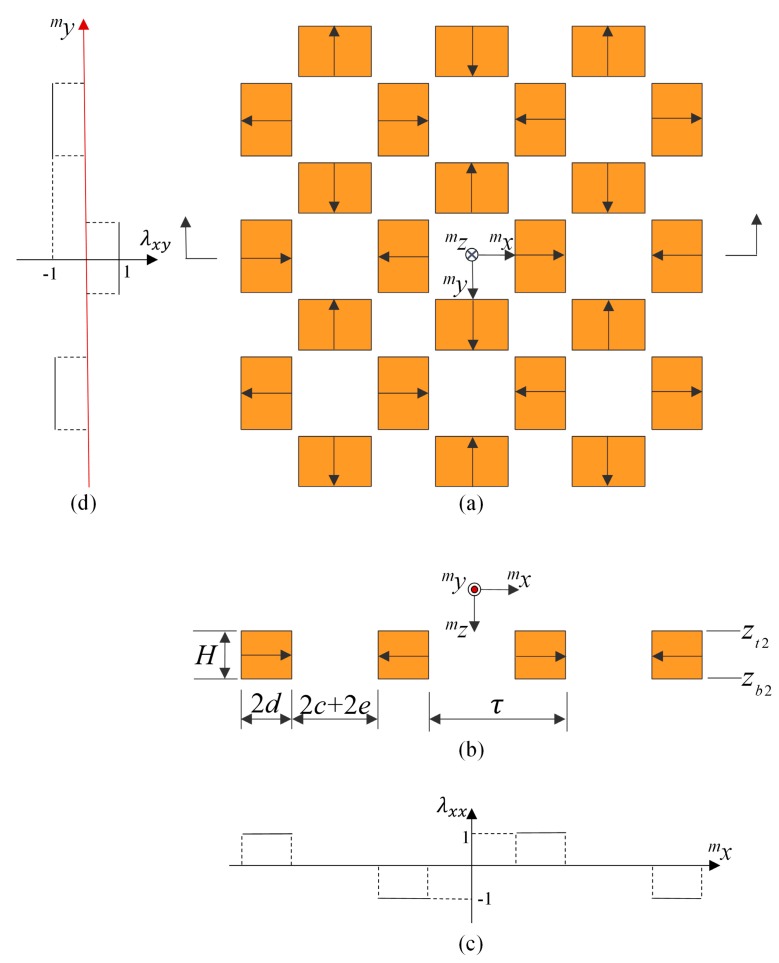
The distribution factors of the residual magnetism amplitude. (**a**) Top view of the magnet array; (**b**) cut-view of the magnet array; (**c**) $\lambda_{xx}$; (**d**) $\lambda_{xy}$.

## Appendix A

The deriving process of the magnetization vector function (taking the sub-array with horizontal magnetized magnets as an example) is listed as follows:

The magnetization vector function describing the magnet array in the coordinate system can be expressed as Equation (A1), and *^m^M_x_*, *^m^M_y_*, and *^m^M_z_* are the components in the *^m^x*-, *^m^y*-, and *^m^z*-directions in Equations (A2)–(A4).

(A1) M→2m=Mxmx^m+Mymy^m+Mzmz^m

(A2) Mxm=λxxλxyM

(A3) Mym=λyxλyyM

(A4) Mzm=0

where $\lambda_{xx}$, $\lambda_{yy}$, $\lambda_{xy}$, and $\lambda_{yx}$ are the distribution factors as shown in Equations (A5) and (A6), and $\lambda_{ij}$ is the *i*-component of the residual magnetism amplitude in the *j*-direction, which are shown in Figure A1.

(A5) $$\lambda_{xx}=\lambda_{yy}=\{\begin{array}{cc} 0 & \left[nT,nT+c+e\right]\\ 1 & \left(nT+c+e,nT+c+e+2d\right)\\ 0 & \left[nT+c+e+2d,nT+3c+3e+2d\right]\\ -1 & \left(nT+3c+3e+2d,nT+3c+3e+4d\right)\\ 0 & \left[nT+3c+3e+4d,nT+4c+4e+4d\right) \end{array}$$

(A6) $$\lambda_{yx}=\lambda_{xy}=\{\begin{array}{cc} 1 & \left[nT,nT+c\right]\\ 0 & \left(nT+c,nT+c+2d+2e\right)\\ -1 & \left[nT+c+2d+2e,nT+3c+2d+2e\right]\\ 0 & \left(nT+3c+2d+2e,nT+3c+4d+4e\right)\\ 1 & \left[nT+3c+4d+4e,nT+4c+4d+4e\right) \end{array}$$

where T=4(c+d+e).

When *n* is large enough, $\lambda_{xx}$, $\lambda_{yy}$, $\lambda_{xy}$, $\lambda_{yx}$ can be expressed as a Fourier series. Thus, the magnetization vector function can be expressed as

(A7) M2→m=Brμ0∑k=1∞∑l=1∞[bkalsinkωx⋅coslωyakblcoskωx⋅sinlωy0]

where $b_{k}=\frac{4}{k\pi }\sin(\frac{k\pi }{2})\sin(k\omega d)$, $a_{l}=\frac{4}{l\pi }\sin(\frac{l\pi }{2})\cos[l\omega (d+e)]$, $a_{k}=\frac{4}{k\pi }\sin(\frac{k\pi }{2})\cos[k\omega (d+e)]$, and $b_{l}=\frac{4}{l\pi }\sin(\frac{l\pi }{2})\sin(l\omega d)$.

## Appendix B

The deriving process of the flux density distribution (taking the sub-array with horizontal magnetized magnets as an example) is listed as follows:

According to Equations (5)–(9), the following equation can be obtained:

(A8) ∇2φ2m=∇·M→mμr=Brωμ0μr∑k=1∞∑l=1∞[(bkalk+akbll)coskωxcoslωy].

Assume that

(A9) φ2m=Brωμ0μr∑k=1∞∑l=1∞[(bkalk+akbll)Z0(zm)coskωxcoslωy]

The following equation can be obtained:

(A10) ∇2φ2m=Brωμ0μr∑k=1∞∑l=1∞[(bkalk+akbll)(d2Z0(zm)dz2−λ2Z0(zm))coskωxcoslωy]

where $\lambda =\sqrt{{\left(k\omega \right)}^{2}+{\left(l\omega \right)}^{2}}$.

In Regions 1 and 3, the general solution of this differential equation is

(A11) Z0(zm)=Cie−λzm+Dieλzm

where *C_i_* and *D_i_* are constants, and *i* = 1, 3.

In Region 2, the general solution is

(A12) Z0(zm)=C2e−λzm+D2eλzm−1λ2.

By using the boundary conditions (Equations (10)–(17)), the value of *C*_1_ and *D*_1_ can be expressed as

(A13) $$C_{1}=0$$

(A14) $$D_{1}=\frac{1}{2\lambda^{2}}\left(e^{-\lambda z_{b2}}-e^{-\lambda z_{t2}}\right).$$

Thus, the following equations can be obtained:

(A15) φ1m=ωBrμrμ0∑k=1∞∑l=1∞[12(e−λzb2−e−λzt2)bkalk+akbllλ2eλzcoskωxcoslωy]

(A16) B→1H=μ0(−∇·φ1m)=12Br∑k=1∞∑l=1∞(e−λzb2−e−λzt2)eλz·[E(k,l)k2+l2ksinkωxcoslωyE(k,l)k2+l2lcoskωxsinlωy−E(k,l)k2+l2coskωxcoslωy]

where $E(k,l)=b_{k}a_{l}k+a_{k}b_{l}l$.
